# α-Linolenic acid increases the G_0_/G_1_ switch gene 2 mRNA expression in peripheral blood mononuclear cells from obese patients: a pilot study

**DOI:** 10.1186/s12944-016-0207-6

**Published:** 2016-02-24

**Authors:** Naiqian Zhao, Li Wang, Na Guo

**Affiliations:** Department of Gerontology, the Second Hospital of Shanxi Medical University, 382 Wuyi Road, Taiyuan, 030001 China

**Keywords:** Obesity, ALA, PPAR-γ, G0S2, GPR 120, FFA, Lipolysis

## Abstract

**Background:**

Recent evidence has demonstrated that the G0/G1 switch gene 2 (G0S2) is an important negative regulator of the rate-limiting lipolytic enzyme adipose triglyceride lipase-mediated lipolysis. It has been revealed that α-linolenic acid (ALA), a plant-based essential omega-3 polyunsaturated fatty acids, reduces adipose tissue lipolysis. However, it is not known whether G0S2 is implicated in ALA-induced inhibition of lipolysis. The purpose of this pilot study is to investigate the effect of ALA on G0S2 gene expression in peripheral blood mononuclear cells (PBMC) of obese patients and the potential influence of G0S2 gene expression in ALA-induced inhibition of lipolysis.

**Methods:**

A total of 26 obese patients were randomly assigned to be treated with or without ALA treatment (~4.0 g daily) for 12 weeks: the ALA-treated group (*n* = 14) or the untreated control group (*n* = 12). Plasma triglyceride (TG), free fatty acids (FFA), glycerol, interleukin-6 (IL-6) and tumor necrosis factor-α (TNF-α), as well as the mRNA expression levels of proliferator-activated receptor gamma (PPAR-γ), G0S2, and G protein-coupled receptor 120 (GPR120) in PBMC were repeatedly examined from fasting obese patients before and after ALA treatment.

**Results:**

ALA significantly decreased plasma TG, FFA, glycerol, IL-6, and TNF-α levels and increased the mRNA expression levels of PPAR-γ, G0S2, and GPR120 in PBMC, compared with the untreated control group. In obese patients from the ALA-treated group, decreased plasma FFA (a biomarker for lipolysis) level was significantly correlated with increased PPAR-γ (a functional omega-3 fatty acids receptor) and G0S2 (a direct target gene of PPAR-γ) mRNA expression in PBMC, while decreased plasma FFA level was not correlated with increased GPR120 (another functional omega-3 fatty acids receptor) mRNA expression in PBMC.

**Conclusion:**

This study shows that ALA increases G0S2 gene expression in PBMC in parallel with the decrease of plasma FFA level in obese patients. Increased G0S2 gene expression might contribute to the beneficial anti-lipolytic effect of ALA in obese patients.

## Background

With the global acceptance of energy-dense foods enriched with saturated fatty acids, the incidence of obesity has dramatically increased throughout the world [1]. In obesity, adipose tissue mass expands by hyperplasia and hypertrophy, and the enlarged adipose tissue releases increasing amounts of free fatty acids (FFA) via increased lipolysis, subsequently resulting in raising plasma FFA [2]. FFA activates the toll-like receptor 4 (TLR4)/the inhibitor kappa B kinase β (IKKβ)/nuclear factor-κB (NF-κB) proinflammatory signaling pathway and induces the expression of proinflammatory cytokines such as interleukin-6 (IL-6) and tumor necrosis factor-α (TNF-α) in a variety of cell types [3–5], thereby contributing to many obesity-related metabolic diseases, including type 2 diabetes, atherosclerotic cardiovascular disease and nonalcoholic fatty liver disease (NAFLD) [6]. Therefore, it is important to reduce lipolysis of the adipose tissue and lower plasma FFA level in the prevention and treatment of obesity-related metabolic diseases.

ω-3 Fatty acids are essential polyunsaturated fatty acids for humans. Nutritionally important ω-3 fatty acids include α-linolenic acid (ALA), eicosapentaenoic acid (EPA), and docosahexaenoic acid (DHA). ω-3 Fatty acids have been shown to improve NAFLD, reduce blood triglyceride levels and incidences of cardiovascular diseases [7–10]. These beneficial effects of ω-3 fatty acids may be due to reduced adipose tissue lipolysis. For example, animal studies have shown that dietary supplementation of EPA and DHA inhibits basal lipolysis and reduces postprandial plasma FFA and glycerol levels in rats fed high-fat diets [11], and that dietary Salba seed rich in ALA improves the impaired anti-lipolytic action of insulin in rats fed high-sucrose diets [12]. In addition, a human study has also demonstrated that dietary fish oil rich in EPA and DHA significantly reduces plasma concentrations of triglyceride and FFA [13].

Considerable evidence has suggested that lipoprotein lipase (LPL) and hormone sensitive lipase (HSL) are involved in the anti-lipolytic effect of ω-3 fatty acids [14]. However, the mechanism(s) by which ω-3 fatty acids reduce adipose tissue lipolysis has not been fully elucidated. The G0/G1 switch gene 2 (G0S2) was initially identified in cultured mononuclear cells during the drug-induced cell cycle switch from G0 to the G1 phase [15, 16]. Recent studies have revealed that G0S2 is a regulator of lipolysis. G0S2 binds directly to the rate-limiting lipolytic enzyme adipose triglyceride lipase (ATGL) and attenuates ATGL-mediated lipolysis via inhibiting the triglyceride hydrolase activity of ATGL in adipocytes [17–19]. Transactivation, gel shift and chromatin immunoprecipitation assays have indicated that G0S2 is a direct proliferator-activated receptor gamma (PPAR-γ) target gene [20]. Interestingly, pharmacological studies have suggested that PPAR-γ is a molecular target of omega-3 fatty acids [21]. Based on these findings, it is tempting to hypothesize that ω-3 fatty acids can increase G0S2 expression, which in turn contributes to ω-3 fatty acids -induced anti-lipolytic effect.

To test this hypothesis, we explored the effects of ALA, an 18-carbon, plant-based essential omega-3 polyunsaturated fatty acids, on the gene expression of PPAR-γ and G0S2 in peripheral blood mononuclear cells (PBMC) of obese patients. In addition, we examined the plasma FFA concentration change in obese patients before and after ALA treatment. Furthermore, we assessed the relationships between changes in plasma FFA concentrations and changes in mRNA expression levels of PPAR-γ and G0S2 before and after ALA treatment in obese patients. For this study, we utilized PBMC to quantify the gene expression changes, as this tissue is approachable in a clinical setting and may reflect a systemic response to ALA treatment.

## Results

26 obese patients were randomly assigned to two groups: the ALA-treated group and the untreated control group. The ALA-treated group included 6 women and 8 men. Among the control obese patients, 5 were women and 7 were men. Table 1 shows a comparison of the clinical and laboratory characteristics between the ALA-treated and control groups before ALA treatment. The two groups were well matched for age, body weight, body mass index (BMI), total cholesterol (TC), triglyceride (TG), FFA, glycerol, IL-6, and TNF-α.

**Table 1:** Clinical and laboratory characteristics before and after ALA treatment

Parameters	Baseline			After 12 weeks		
	ALA (*n* = 14)	Control (*n* = 12)	p	ALA (*n* = 14)	Control (*n* = 12)	p
Age (years)	48.6 ± 6.8	49.3 ± 4.8	0.775			
Body weight (kg)	93.6 ± 6.6	95.3 ± 6.6	0.329	89.8 ± 5.8	92.6 ± 6.7	0.265
BMI (kg/m^2^)	32.3 ± 1.2	32.7 ± 1.0	0.331	31.0 ± 1.3	31.8 ± 0.8	0.076
TC (mmol/L)	5.01 ± 0.48	5.17 ± 0.45	0.418	4.79 ± 0.42	5.01 ± 0.41	0.203
TG (mmol/L)	3.59 ± 0.65	3.73 ± 0.63	0.565	1.73 ± 0.31	2.63 ± 0.54	<0.0001*
FFA (μmol/L)	655 ± 324	662 ± 28	0.516	469 ± 29	501 ± 28	0.009*
Glycerol (μmol/L)	1505 ± 27	1538 ± 83	0.183	1028 ± 82	1201 ± 94	0.046*
IL-6 (ng/L)	2.34 ± 0.14	2.37 ± 0.15	0.603	1.53 ± 0.14	1.87 ± 0.12	<0.0001*
TNF-α (ng/L)	1.92 ± 0.14	1.92 ± 0.16	0.981	1.28 ± 0.10	1.63 ± 0.15	<0.0001*

Data are means ± SD

ALA vs Control, two-sample t test, *p < 0.05

After ALA treatment for 12 weeks, BMI and plasma concentrations of TG, FFA, glycerol, IL-6, and TNF-α were significantly lower in obese patients from the ALA-treated and control groups compared with before ALA treatment. There were no significant differences in body weight and TC in obese patients from the ALA-treated and control groups between after and before ALA treatment (Table 2).

**Table 2:** Change of clinical and laboratory characteristics before and after ALA treatment

Parameters	ALA (*n* = 14)			Control (*n* = 12)		
	Baseline	After 12 weeks	p	Baseline	After 12 weeks	p
Age (years)	48.6 ± 6.8			49.3 ± 4.8		
Body weight (kg)	93.6 ± 6.6	89.8 ± 5.8	0.116	95.3 ± 6.6	92.6 ± 6.7	0.335
BMI (kg/m^2^)	32.3 ± 1.2	31.0 ± 1.3	0.009*	32.7 ± 1.0	31.8 ± 0.8	0.018*
TC (mmol/L)	5.01 ± 0.48	4.79 ± 0.42	0.209	5.17 ± 0.45	5.01 ± 0.41	0.381
TG (mmol/L)	3.59 ± 0.65	1.73 ± 0.31	<0.0001*	3.73 ± 0.63	2.63 ± 0.54	0.048*
FFA (μmol/L)	655 ± 324	469 ± 29	<0.0001*	662 ± 28	501 ± 28	<0.0001*
Glycerol (μmol/L)	1505 ± 27	1028 ± 82	<0.0001*	1538 ± 83	1201 ± 94	<0.0001*
IL-6 (ng/L)	2.34 ± 0.14	1.53 ± 0.14	<0.0001*	2.37 ± 0.15	1.87 ± 0.12	<0.0001*
TNF-α (ng/L)	1.92 ± 0.14	1.28 ± 0.10	<0.0001*	1.92 ± 0.16	1.63 ± 0.15	<0.0001*

Data are means ± SD

Baseline vs After 12 weeks, two-sample t test, *p < 0.05

Table 1 also shows a comparison of the clinical and laboratory characteristics between the ALA-treated and control groups after ALA treatment for 12 weeks. Plasma concentrations of TG, FFA, glycerol, IL-6, and TNF-α were significantly lower in obese patients from the ALA-treated group compared with the control group. There were no significant differences in body weight, BMI, and TC in obese patients between the two groups.

As mentioned above, PPAR-γ functions as a receptor for omega-3 fatty acids and stimulates the expression of G0S2 gene that is involved in lipolysis. G protein-coupled receptor 120 (GPR120) is another functional omega-3 fatty acids receptor and mediates the potent anti-inflammatory effect of omega-3 fatty acids, leading to the inhibition of the NF-κB pathway, which then inhibits the production of many proinflammatory cytokines including IL-6 and TNF-α [22]. In order to describe the ALA-related intracellular function alterations, the mRNA expression levels of PPAR-γ, G0S2, and GPR120 in PBMC were measured. Before ALA treatment, there were no appreciable differences in the mRNA expression levels of PPAR-γ, G0S2, and GPR120 between the two groups (Table 3). Treatment with ALA for 12 weeks caused a significant increase of the mRNA expression levels of PPAR-γ, G0S2, and GPR120 in obese patients from the ALA-treated group (Table 4). There were no significant differences in the mRNA expression levels of PPAR-γ, G0S2, and GPR120 in the control group between after and before ALA treatment (Table 4). Compared with the control group, treatment with ALA for 12 weeks caused a significant increase of the mRNA expression levels of PPAR-γ, G0S2, and GPR120 (Table 3).

**Table 3:** mRNA expression of PPAR-γ, G0S2, and GPR120 before and after ALA treatment

	Baseline			After 12 weeks		
	ALA (*n* = 14)	Control (*n* = 12)	p	ALA (*n* = 14)	Control (*n* = 12)	p
PPAR-γ	1.16 ± 0.16	1.22 ± 0.18	0.330	2.29 ± 0.11	1.25 ± 0.13	<0.0001*
G0S2	1.08 ± 0.15	1.12 ± 0.15	0.564	2.25 ± 0.12	1.17 ± 0.13	<0.0001*
GPR120	1.06 ± 0.16	1.11 ± 0.15	0.411	1.70 ± 0.12	1.18 ± 0.16	<0.0001*

Data are means ± SD

ALA vs Control, two-sample t test, *p < 0.05

**Table 4:** Change of mRNA expression of PPAR-γ, G0S2, and GPR120 before and after ALA treatment

	ALA (*n* = 14)			Control (*n* = 12)		
	Baseline	After 12 weeks	p	Baseline	After 12 weeks	p
PPAR-γ	1.16 ± 0.16	2.29 ± 0.11	<0.0001*	1.22 ± 0.18	1.25 ± 0.13	0.657
G0S2	1.08 ± 0.15	2.25 ± 0.12	<0.0001*	1.12 ± 0.15	1.17 ± 0.13	0.323
GPR120	1.06 ± 0.16	1.70 ± 0.12	<0.0001*	1.11 ± 0.15	1.18 ± 0.16	0.330

Data are means ± SD

Baseline vs After 12 weeks, two-sample t test, *p < 0.05

**Fig. 1:**
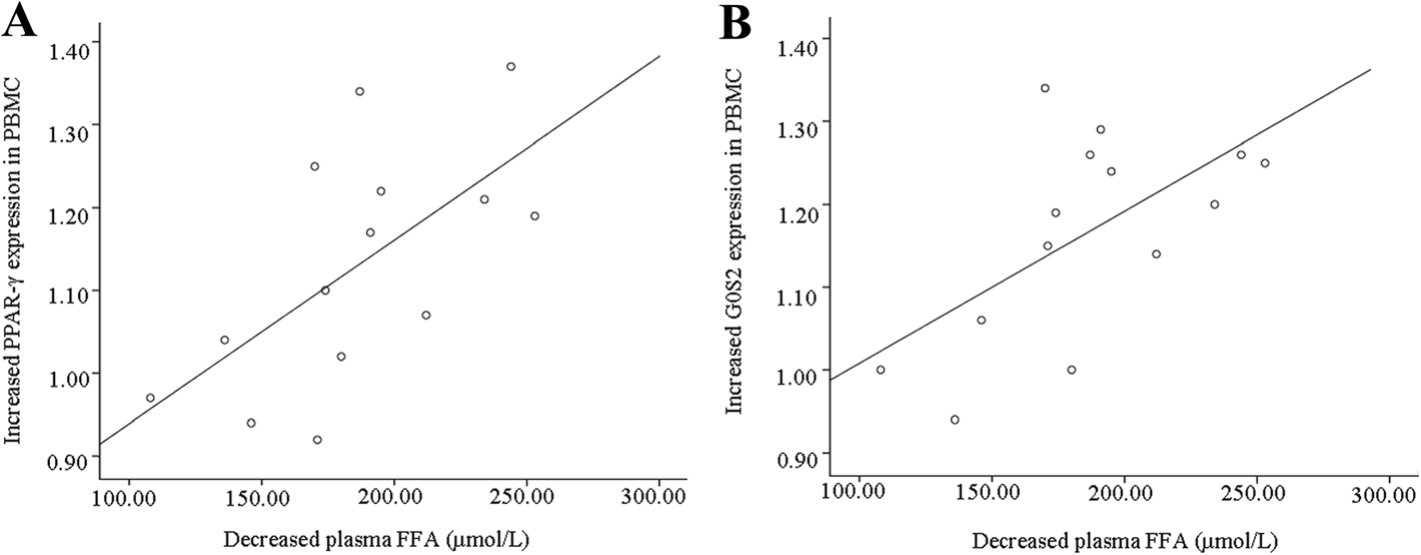
Correlation between decreased plasma FFA concentration and **a** increased PPAR-γ (*n* = 14; *r* =0.634, *P* = 0.015) or **b** G0S2 (*n* = 14; *r* = 0.613, *P* = 0.020) mRNA expression in PBMC in obese patients from the ALA-treated group

During lipolysis in adipocytes, TG is hydrolyzed into fatty acids and glycerol. Fasting plasma FFA concentration is used as a parameter for lipolysis, because in the fasting state, plasma FFA arise almost entirely from hydrolysis of TG in adipocytes [23]. To gain some mechanistic insights, we undertook different correlations between changes in plasma FFA concentrations and changes in mRNA expression levels of PPAR-γ and G0S2 before and after ALA treatment in obese patients from the ALA-treated group. Decreased plasma FFA concentration was significantly correlated with increased PPAR-γ (*r* =0.634, *P* = 0.015) and G0S2 (*r* = 0.613, *P* = 0.020) mRNA expression in PBMC (Fig. 1). We also examined whether increased GPR120 mRNA expression in PBMC was associated with increased PPAR-γ and G0S2 mRNA expression in PBMC, and decreased plasma FFA concentration. In obese patients from the ALA-treated group, increased GPR120 mRNA expression in PBMC was not correlated with increased PPAR-γ (*r* = 0.453, *P* = 0.104) and G0S2 (*r* = 0.484, *P* = 0.080) mRNA expression in PBMC, and decreased plasma FFA concentration (*r* = 0.229, *P* = 0.431). Since TNF-α can stimulate basal lipolysis in adipocytes [24], we further correlated decreased plasma FFA concentration with decreased plasma TNF-α concentration to test whether decreased plasma TNF-α concentration was implicated in a potential antilipolytic effect of ALA. In obese patients from the ALA-treated group, decreased plasma FFA concentration was not correlated with decreased plasma TNF-α (*r* = 0.283, *P* = 0.327) concentration.

## Discussion

Adipose tissue controls plasma FFA levels by modulating the release of FFA into the circulation. Obesity is associated with increased lipolysis, which leads to excessive release of FFA into the circulation thereby contributing to the development of a cluster of chronic metabolic disorders, including type 2 diabetes and atherosclerotic cardiovascular disease. Thus, manipulation of inhibiting lipolysis is a logical means of alleviating these obesity-related chronic metabolic diseases. In a rodent study, intake of dietary Salba seed rich in ALA for 3 months reversed the impaired anti-lipolytic action of insulin and dyslipidemia of rats fed a sucrose-rich diet [12]. In this study, we found that 12- week treatment with ALA significantly decreased plasma TG levels in obese patients. This result is consistent with the clinical observation that the ALA intake of 3.5 g/d for 3 months was effective in reducing plasma TG levels in patients with the metabolic syndrome [25]. Specifically, we confirmed that ALA led to a pronounced reduction in plasma FFA concentrations, a biomarker for lipolysis, in obese patients. These findings have indicated that ALA inhibits adipose tissue lipolysis.

The mechanisms for adipocytes lipolysis process are complex. Lipolysis is mediated by cytosolic lipases that catalyze TG hydrolysis in adipocytes. The rate-limiting and the most important lipase of TG hydrolysis is ATGL [26–31]. ATGL is a widely expressed enzyme that is responsible for hydrolyzing TG into diacylglycerol and FFA. G0S2 is an important negative regulator of ATGL-mediated lipolysis and suppresses ATGL activity in a dose-dependent manner [17]. In cultured mouse adipocytes, PPAR-γ agonist rosiglitazone significantly increased G0S2 protein expression [17], and the G0S2 promoter encompasses a PPAR-responsive element (PPRE) [20], showing that G0S2 is a direct target gene of PPAR-γ. As PPAR-γ is a molecular target of ALA [21], we quantitated gene expression levels of PPAR-γ and G0S2 in PBMC of obese patients before and after ALA treatment to test whether PPAR-γ and G0S2 gene expression are affected by ALA. We found that ALA significantly increased gene expression levels of PPAR-γ and G0S2 in PBMC of obese patients. Taken together, these results have suggested that ALA induces the upregulation of G0S2 gene expression in PBMC potentially in part through the activation of PPAR-γ. However, further studies are necessary to elucidate the causal relationship between an increase of PPAR-γ and G0S2 gene expression in PBMC by ALA. In addition, this study also revealed that increased PPAR-γ and G0S2 mRNA expression levels in PBMC were significantly correlated with the reduction of plasma FFA concentrations from before to after ALA treatment. Given that changes in G0S2 mRNA expression in PBMC likely reflect corresponding changes in G0S2 mRNA expression in adipose tissue and that G0S2 plays an important role in inhibiting adipose tissue lipolysis, our results have suggested that the effect of ALA on G0S2 mRNA expression might be attributable to anti-lipolytic property of ALA. However, further investigation is required to clarify whether ALA induces G0S2 gene expression in adipose tissue, thus contributing to reducing lipolysis.

GPR120, officially described as free fatty acid receptor 4 (FFAR4), is a functional omega-3 fatty acids receptor highly expressed in proinflammatory macrophages, and omega-3 fatty acids exert their anti-inflammatory effects through this receptor [22]. In this study, we found that 12-week treatment with ALA significantly increased GPR120 gene expression of PBMC and decreased plasma IL-6 and TNF-α levels in obese patients. Our results are compatible with a recent report [32], showing that flaxseed oil enriched in ALA significantly increased colonic FFAR4 expression and reduced expression of the pro-inflammatory cytokine TNF-α. In cultured mouse adipocytes, EPA upregulated vascular endothelial growth factor-A (VEGF-A), a known PPAR-γ target gene, mRNA expression by inducing PPAR-γ binding to PPRE in VEGF-A promoter region [33]. Furthermore, transfection of GPR120 gene enhanced EPA-induced PPAR-γ binding to PPRE in VEGF-A promoter leading to increased VEGF-A mRNA expression [33]. To evaluate the influence of GPR120 on anti-lipolytic property of ALA, we analyzed the correlations between changes in GPR120 mRNA expression and changes in PPAR-γ and G0S2 mRNA expression, changes in plasma FFA concentration before and after ALA treatment. We showed that changes in GPR120 mRNA expression were not correlated with changes in PPAR-γ and G0S2 mRNA expression, and changes in plasma FFA concentration. Some studies have reported that TNF-α is related to the stimulation of adipose lipolysis in obesity [25]. To test whether ALA exerts its anti-lipolytic effect by inducing a decrease in plasma TNF-α level, we then analyzed the correlation between decreased plasma FFA concentration and decreased plasma TNF-α concentration. We also showed that decreased plasma FFA concentration was not correlated with decreased plasma TNF-α concentration. Given that the sample size was too small and that correlation studies don’t establish a direct causal relationship, we were not completely sure that GPR120 does not play a role in modulating G0S2 gene expression and that decreased TNF-α level is not implicated in the anti-lipolytic effect of ALA.

In addition, although we observed that there was a tendency for lower body weight and BMI in obese patients following ALA treatment, we did not specifically measure body fat composition. Thus, we were not able to determine the degree of correlation between changes in plasma FFA concentration and changes in body fat composition. Together, we observed changes in PPAR-γ and G0S2 gene expression in PBMC correlated with changes in plasma FFA concentration, implying that G0S2 might mediate the anti-lipolytic effect of ALA.

There are several limitations in the current study. First, because the small sample size is inadequate to generate conclusive data, it is necessary to conduct a large sample prospective cohort study in order to clarify the involvement of G0S2 in PBMC in the improvement of obesity-related impaired lipolysis by the treatment with ALA. Second, owing to correlation analyses of this study, causal relationships between PPAR-γ and G0S2 gene expression, and between G0S2 gene expression and reduced lipolysis could not be established. Thus, a mechanistic study is needed to elucidate the effect of PPAR-γ on G0S2 gene expression and the effect of G0S2 gene expression on reduced lipolysis by the treatment with ALA. Third, the measurement of gene expression in PBMC might not accurately indicate gene expression changes in adipose tissue following the treatment with ALA. However, obtaining adipose tissue is invasive and therefore is not clinically feasible to perform for research purpose. Thus, further examination is required to investigate whether adipose tissue is consistent with PBMC in PPAR-γ and G0S2 gene expression changes following the treatment with ALA.

In conclusion, this study demonstrated that ALA increased PPAR-γ and G0S2 gene expression in PBMC in parallel with the decrease of plasma FFA levels in obese patients. Although it is not possible to conclude with surety that these results are predictive of the lipolysis status in adipose tissue, given the anti-lipolytic property of G0S2, the beneficial anti-lipolytic effect of ALA may be due, at least in part, to increased G0S2 expression in adipose tissue. As ALA has been proven to reduce plasma triglyceride levels [25], blood pressure [34], and the risk of cardiovascular disease and type 2 diabetes [35], the results of this study provide important insights into its therapeutic implications in obesity-related metabolic disorders.

## Methods

### Subjects and study protocol

During the period from June 2014 to January 2015, we recruited 26 obese patients through the outpatient clinic at the Second Hospital of Shanxi Medical University in China. All obese patients were with a BMI of > 30.0 kg/m^2^ and had stable body weight for 6 months before the study. Other inclusion criteria were blood pressure < 140/90 mmHg, TC <6.0 mmol/L, TG >1.7 mmol/L, fasting plasma glucose <6.1 mmol/L and 2-hour plasma glucose after a 75 g oral glucose load <7.8 mmol/L. Patients with the following indications were excluded from the study: severe renal diseases, severe liver dysfunction, acute or chronic inflammatory disease, autoimmune disease, steroid therapy, anti-inflammatory drugs therapy, statin and other hypolipidemic therapy. This study was a prospective, randomized, open-label design, using simple randomization. 26 obese patients were assigned to the ALA-treated and control groups. The ALA-treated group received four perilla oil soft capsules (2.0 g) containing highly purified (≥95 %) ALA (Fujian Sanai Pharmaceutical Co., Ltd., Fuzhou, China) twice a day [34] combined with individual lifestyle counseling by a physician, with emphasis on diet and exercise [36] for 12 weeks. The control group received the same individual lifestyle counseling for 12 weeks. The daily dose of ~ 4.0 g ALA was based on several clinical trials [35]. The study protocol was approved by the Medical Ethics Committee at the Second Hospital of Shanxi Medical University. The procedures followed were conducted in accordance with the principles of the Declaration of Helsinki. Written informed consent was obtained from all patients.

### Data collection

The patients underwent a medical history, physical examination, and screening laboratory tests. At the beginning and end of the study, blood samples were obtained in the morning after an overnight fast. We measured plasma levels of TC, TG, FFA, glycerol, IL-6, and TNF-α. Human PBMC were isolated from venous blood using Ficoll-Hypaque density gradient centrifugation. We analyzed the expression of PPAR-γ, G0S2, and GPR120 mRNA in PBMC using a real-time quantitative PCR method.

### Preparation of PBMC

After overnight fasting, 30 ml venous blood was drawn into an EDTA-coated vacutainer tube. The blood was diluted two-fold with phosphate buffered saline (PBS, pH 7.4). 4 ml diluted blood was layered onto 4 ml Ficoll-Hypaque Plus solution (Amersham Pharmacia Biotech AB, Sweden) and centrifuged at 2000 rpm for 20 min at room temperature. PBMC were removed from the plasma-Ficoll interface and washed three times in PBS to remove platelets, Ficoll-Hypaque and plasma. Collected PBMC were cryopreserved at -80 °C and used later for quantification of mRNA expression.

### Quantitative real-time PCR

Total RNA was isolated from PBMC using TRIzol reagent (Takara Bio, Shiga, Japan) according to the manufacturer’s instructions, and RNA quality was evaluated via electrophoresis. Reverse transcription (RT) was performed using the PrimeScrip RT reagent Kit (Takara Bio, Shiga, Japan). The RT conditions for each cDNA amplification were 37 °C for 15 min, 85 °C for 5 s, and 4 °C for ∞. Gene expression analysis was performed by quantitative PCR (qPCR) on a StepOnePlus^TM^ Real-Time PCR System (Applied Biosystems, Inc., Foster City, CA, USA) using SYBR green as the detection dye. Primer sequences used for the detection of genes were designed as follows: PPAR-γ forward primer: 5’-ATGACAGACCTCAGACAGA-3’; reverse primer: 5’-AATGTTGGCAGTGGCTCACG-3’; G0S2 forward primer: 5’-ACCACAAGCATCCACCAA-3’; reverse prime: 5’-GCATTTATCCTTCCTCCCTA-3’; GPR120 forward primer: 5’- AGCCACCAGATCCGCGTGT-3’; reverse primer: 5’- TTGAAGTTCTGGATCAGGA-3’; glyceraldehydes 3-phosphate dehydrogenase (GAPDH) forward primer: 5’-CCCACTGCCAACGTGTCA-3’ and reverse primer: 5’-AAGTCAGAGGAGACCACCT-3’. The expected size of the amplified product was 148 bp (PPAR-γ), 135 bp (G0S2), 131 bp (GPR120) and 152 bp (GAPDH). GAPDH was used as a control housekeeping gene. Cycling conditions were 95 °C for 30 s and 95 °C for 5 s, followed by 40 cycles at 64 °C for 34 s. The predicted sizes of the PCR products were confirmed by 2 % agarose gel electrophoresis stained with ethidium bromide. Melting curve analysis was performed for each sample in direct connection to the PCR, to verify the purity of the amplified PCR product. The results were stated as the fold difference expression for each target gene compared to that of GAPDH as an internal control in the same sample, using the 2^−ΔΔCt^ method. All samples were performed in duplicate.

### Laboratory analyses

Plasma levels of TC and TG were measured in EDTA-plasma using routine methods at the central laboratory at the Second Hospital of Shanxi Medical University (Taiyuan, China). Lipolysis was evaluated by measuring plasma levels of glycerol and FFA using enzyme-based colorimetric assay kits according to the manufacturer’s instructions (Randox, UK). The detection limits were 1 μmol/L glycerol and 2 μmol/L FFA. Intra-assay and inter-assay coefficient of variation (CV) for glycerol and FFA were <10 %. Plasma IL-6 and TNF-α levels were determined using ELISA kits following the manufacturer’s instructions (R&D Systems, USA). The detection limits were 0.70 ng/L IL-6 and 0.5 ng/L TNF-α. Intra-assay and inter-assay CV for IL-6 and TNF-α were <6 % and <8 %, respectively. Every sample was analyzed in duplicate.

### Statistical analysis

All data are expressed as mean ± SD. Two-sample t tests were used for comparisons of the means between the two groups at the baseline or posttreatment and those between the baseline and posttreatment in the two groups. Pearson correlation coefficient was employed to investigate the correlations between changes in plasma FFA concentrations and changes in mRNA expression levels of PPAR-γ and G0S2 and those between changes in GPR120 mRNA expression level and changes in plasma FFA concentrations and mRNA expression levels of PPAR-γ and G0S2 before and after ALA treatment in obese patients from the ALA-treated group. Differences were considered statistically significant at *p* < 0.05. All analyses were performed using SPSS version 16.0 (IBM, New York, USA).

## Abbreviations

ALA: α-linolenic acid
ATGL: adipose triglyceride lipase
BMI: body mass index
CV: coefficient of variation
DHA: docosahexenoic acid
EPA: eicosapentaenoic acid
FFA: free fatty acids
FFA4: free fatty acid receptor 4
GAPDH: glyceraldehydes 3-phosphate dehydrogenase
G0S2: G0/G1 switch gene 2
GPR120: G protein-coupled receptor 120
IL-6: interleukin-6
NAFLD: nonalcoholic fatty liver disease
NF-κB: nuclear factor-κB
PBMC: peripheral blood mononuclear cells
PPAR-γ: proliferator-activated receptor gamma
PPRE: PPAR-responsive element
RT: reverse transcription
TC: total cholesterol
TG: triglyceride
TNF-α: tumor necrosis factor-α
VEGF-A: vascular endothelial growth factor-A
